# Editorial

**Published:** 1851-07

**Authors:** 


### AMERICAN MEDICAL ASSOCIATION AND DENTAL COLLEGES.

The American Medical Association has been formed as we have always supposed for the promotion of Medical and Surgical science—having for its object the diffusion of knowledge, and incidentally the promotion of good feeling among those engaged in its study and practice. A time honored profession thus uniting for a noble purpose should and does command our highest admiration. This feeling must if possible be heightened, when we see the fathers in the profession, those who have labored for a long life in its arduous duties, travelling hundreds of miles to participate in the discussions, freely imparting the experience of years, and asking nothing in return but a reciprocal confidence.

The constitution of the Association we believe guards with commendable propriety the admission of delegates. These must all be graduates, and that too of regular medical schools—we might it is true ask, why make a distinction invidious to Dental Colleges. We believe that such should not be the case, but as this question can hereafter be discussed, we will for the present take up one forced upon us by a resolution offered at the last meeting of the American Medical Association. The minutes to which I have access give the following report, by Dr. Wood, of Pennsylvania:—"Resolved, that Colleges exclusively of Dentistry and Pharmacy, are not recognized by the association as among the bodies authorized to send delegates to its meetings."

Dr. Wood of New York moved to amend, by dividing the resolution, so as to take the question, first, on the reception of delegates from Colleges of Dentistry. Secondly on the reception of delegates from Colleges of Pharmacy.

The amendment being accepted, the reception of delegates from Colleges of Dentistry was debated.

Dr. Lamb moved an indefinite postponement of the resolution, which was lost.

A motion was "finally made by Dr. Hays of Pennsylvania, that the whole resolution of Dr. Wood, including Colleges of Dentistry and Pharmacy, be referred to a special committee of five members, which resolution was adopted." We have thus presented the action of the association on this very important subject—we say important because all that relates to medical Science must be so viewed, and because the resolution aims a blow at one of the most important specialities of medical and surgical science—this has been done too, by one occupying a very prominent position in the medical profession—Professor Wood of the University of Pennsylvania.

The resolution would seem to say that none but those practicing or teaching all the different specialities which go to make up medical science, should be entitled to a seat in the association. Yet this cannot be the design of the mover of the resolution, for we have never heard that Prof. Wood, either teaches or practices Dental Surgery, if so he has failed to acquire a reputation in either. Many of our best Physicians devote almost their entire attention to some particular speciality of medicine. Indeed we look around upon the profession and find many who have distinguished themselves, but scarce one who excel in more than one department—so much is this the case, that it is not unusual for those in our larger cities to exclusively aim at a certain kind of practice—hence we have our occulists, aurists, surgeons, obstetricians, &c. &c. In Europe this division becomes more perfect, and the observation of every one would say, that if we wish to excel in any department of science, our studies must be mainly devoted to that branch.

Our Dental Colleges have been instituted to supply a deficiency which has ever existed in our medical schools, and which with the now advanced state of Dental science cannot be supplied in any other way. It would appear strange that now when it is generally conceded that for the amount of instruction which should be given in our medical schools, the session should be lengthened, that an effort should be made to discredit dental instruction, by means of Dental Institutions, and thus if possible bring into the medical classes, the few but increasing number which make up the dental.

But what have the Dental Colleges done that they should be thus excluded? They have combined in this course of instruction to meet the wants of the Dental practitioner, a pretty thorough course in the study of Anatomy, believing that the dentist should know something more than the structure of the maxillary bones, or even the origin and distribution of those nerves which supply the teeth with sensation, that indeed without a knowledge of the general organization they cannot understand the sympathies manifested through dental irritation, or the diseases which are aggravated and depend on this cause. They have also thought it a duty to demand of the dental student a knowledge of Pathology and Therapeutics, that they may be enabled to investigate and account for diseased action and Physiology, that they may be better enabled to understand the former. They have also thought it necessary to teach the general principles of chemical science, and especially that part which teaches the constituents of tooth bone and the agents which may injure these important organs. We believe that the dentist should be able to direct the parent in that course of diet which might secure to the child the best possible osseous system, and hence the best constituted teeth. Thus far they have attempted to secure to the Dental practitioner, a basis on which he may confidently expect to make himself useful to the public, and a worthy co-laborer in that science which it is and should be his ambition to advance, and of which he claims to be a representative.

The above studies are thought important—Prof. Wood cannot regard them as more so, and we believe that in their application to dental practice, they can be better taught to a dental class, and by one who makes it his study to some extent, than in any of our medical schools. All the Chairs in our dental schools, except those strictly dental, are occupied by medical gentlemen of the first standing, and who would fill the same chairs reputably in any of the medical schools of the land.

We had intended giving insertion to Dr. Gardeth's proposition to establish a Professorship of Dental Surgery to the several medical faculties, with some comments thereon in the present number of the Register, but for want of space it must be defered to the next number—we shall then have something more to say on the resolution of Dr. Wood.

---

Contributions to the Library and Museum of the Ohio College of dental Surgery.—Dr. Allen of New York has very liberally sent us for the Library of the College, a complete set of the Recorder. Messrs. Jones, White & Co. have also furnished us with the Dental News Letter complete, for the same purpose. These donations we regard as invaluable, for we expect our students to be more interested and profited in the latest publications and improvements of the profession, than in that which has long since been superceded by the advanced state of the science.

We have just received from our friend, Dr. Samuel B. Fithian, of St. Louis, a valuable contribution to the Anatomical cabinet of the Dental College.

It consists of some rare osseous specimens, illustrating the strange freaks that nature sometimes makes in the development of the teeth, together with a perfect and beautiful *Ethmoid* bone—several bones of the foetal cranium, and a set of artificial teeth, carved from a tusk of the sea horse. The last being the representative of a past dental era, is singularly valuable, as showing the great improvements that have been made in the science of dentistry since the time of their construction.*

Two of the specimens showing the derangements in the growth of the teeth are so rare and beautiful as to be worthy of a special description.

The first is an upper jaw containing nineteen teeth, arranged in a very peculiar manner. The bicuspids and molars are regular in number and position, but in the alveolar process anterior to the first bicuspids, nine teeth are crowded; standing in three rows. The anterior row contains four teeth—*the two central incisors, and two cuspids.* The second row is made up of *two anomalous*

* This set was constructed by the renowned Fox, of London, and was worn some six years and cost five hundred dollars.

teeth and one *lateral incisor*—and the posterior row of one anomalous tooth, and one lateral incisor. The whole of the teeth in the jaw are of the largest size of the classes to which they belong. The incisors and cuspids are unusually large, which, together with the anomalous teeth require a great antero-posterior extension of the alveolar process in which they are planted. This extension is near three quarters of an inch.

Two of the anomalous teeth have a very peculiar shape. Their roots are similar to those of the incisors, but their bodies have the appearance of having been originally formed like the incisors, with a broad cutting edge, but which in its development has been coiled upon itself like a scroll, making rather more than one complete turn, having one extreme of the cutting edge in the centre and the other on the periphery of the tooth.

The other anomalous tooth resembles a cuspid, though from its confined position it is the smallest tooth in the jaw. Its germ appears to have originated in the socket of the first biscuspid of the right side, but taking a horizontal direction in its development, has insinuated itself between the anterior and posterior rows, and on a line with a middle row. Its root is against the root of the biscuspid, while the apex of its crown extends across the maxillary suture and is in contact with the root of the anomalous tooth of the left side, the tooth lying in such a position as to have been entirely buried beneath the gum in the living person.

The second specimen is a superior maxilla in which the ridge corresponding with the alveolar processes have no marks of teeth, except at one point, and that is at the space usually occupied by the cuspid of the right side. On the left side is a well developed cuspid tooth completely imbedded in the bone. It lies horizontally, extending from the middle of the canine fosa to the *foramen incissivum*, in which the point of its crown is visible.

It is covered above by the meatus nares, below by the spire of bone that is formed by the imperfectly developed alveoli, posteriorly by the palatine process, and before by a thin lamen of bone, which has been cut away, so as to expose the tooth and the cavity where it is imprisoned.

We have from Dr. Sanford, Chillicothe, Ohio, a beautiful case of a broken front incisor, broken just at the neck and turned inwards and united at right angles with the root—the fracture is distinct and the reunion perfect.

We have also from Dr. Locke, Hamilton, Ohio, a beautiful specimen in plaster model, of tranposition of the incisors above, the lateral taking the place of the front.

We have also recently saved in our own practice a few of the largest human teeth we ever saw; one not enlarged in the roots by disease; two extraordinarily enlarged by exostosis. We hope the profession will preserve such specimens, and we would be glad to have as many deposited in the Museum of the College as we can get.

---

Filling teeth after the Lining Membrane has become exposed.—We are glad to see this subject attracting so much attention, and we are confident that many teeth are preserved for years, which only a few years since would have been consigned to the forceps. A living tooth we regard as always better than a dead one, and hence the importance of preserving its vitality. We expected to have prepared for the present number of the Register, an article on this subject, but have been obliged to defer it. We are, however, keeping notes of cases on this subject, and the success thus exhibited is far more gratifying than was anticipated. Dr. Van Emon, also informs us that such has been the case in his practice. We are satisfied that ossification of the pulp, often takes place after the teeth has been filled. If the irritation occurs after the operation becomes extensive, the death of the pulp will generally take place, but this does not always result in abscess and sometimes no special soreness of the teeth. This, however, would be a favorable case—slight irritation may induce a deposit of bone, and we had a case of this kind, treated two years since. The teeth were anterior and posterior molars, left side inferior maxillae ; the teeth both had been painful and this from a mere irritation of decay—the decay was gradually at different times removed, and articles used to subdue irritation, and at the end of ten days teeth filled, since which they have done well. Before filling I found the pulp cavity shielded by deposit of bone, and the nerve evidently alive and such I believe to be the still case. We regard therefore the practice of Dr. W. W. Codman, of Boston, as one which will often prove successful. The habits, constitution and temperament of the patient will have, we think much to do in the success of the operation.

---

Dunlevy's Gold Foil.—In a former number of the Register, we spoke of this article, since which time we have been more closely testing its quality. There are two especial requisites, which we regard as essential in gold foil for our use, perhaps the most important is softness. Foil which is hard and brittle cannot be used to advantage in filling teeth, and this is especially the case in small and difficult cavities, and with such foil there is a constant waste, for the scraps and portions which have been ruffled by handling cannot be used. The softer the foil the more readily will it yield to the pressure of the instrument, and hence more perfectly adapts itself to the inequalities of the cavity, and when soft it is nearly always tough.

The second requisite is adhesiveness,—to some this may not seem essential, yet for filling with blocks it is highly requisite. Foil when folded should adhere together, and when rolled into blocks unless it does adhere, the blocks will be coming to pieces and trouble very much the operator.

These qualities we have found in Mr. Dunlevy's foil, and these we regard as the essentials which have made Mr. Abbey's foil so justly celebrated. In connection with this we have as in Abbey's—substance ; it is not tinsel like, requiring the care in handling which is necessary in leaf. It bears a fine finish, and if the profession wish to know more, they can get it at Dr. Brown's Dental Depot, Cincinnati, or of Mr. Dunlevy himself, Pittsburgh, who will at all times attended with promptness to all orders remitting the cash.

---

American Society of Dental Surgeons.—This society will hold its twelfth annual meeting in Philadelphia on the 5th of August next. We hope there may be a large attendance, and doubt not the discussions will be of an inter esting and instructive character.

DENTAL LATHE.—We have a very neat and convenient Lathe gotten up by Dr. Buckwell, of Zanesville, and which answers an admirable purpose. The upright to which the cylinder head and chuck for mandrels are attached is about eight inches high, so arranged as to be fastened by screw to a table or bench. The wheel for the cord is turned with the cylinder and is about one and a half inches in diameter, which with a moderate sized tread wheel gives great velocity. The upright stock has about four inches from its base, a bar extending out some six inches, to which is attached a moveable rest, so arranged as to be changed to any position desired. These are made of brass, also, a neat cap which cover the cylinder head to keep out the dirt. We have some doxen mandrels for Emery wheels, drill, &c. The Emery wheels are secured by a small screw tap, and the Lathe run by treddle, with a cat gut or gutta percha cord. This Lathe forms for an operating room the neatest article of the kind we have ever seen, and, excepting the tread wheel and cord, can be had for about fifteen dollars. A very neat and accurate workman in Zanesville will make them for that price.

---

"Ivery Friday I will be to home the whole day to clean, plug and set teeth. Plenty of the same stuff on hand, one drop will stop it in ten or fifteen minutes if well applied."

"The above is an exact copy of a written card found tacked to a gate post five miles from this place and I understand they are to be seen at various cross roads, public houses, &c. This boy as you will see by this card is one of them, but he is not the only one that can be found through our country."

The above card was sent us by a friend, and is only a specimen of what we occasionally meet with throughout the whole country—to the above was attached an M. D. It is not worse however than much we meet with coming from those claiming high positions in the profession. Were we to publish the card of some, we have no doubt they would be highly offended.

It is degrading, disgusting and disreputable to see old practitioners trickering and puffing themselves into notice and this often at the expense of truth and honesty.

---

THE MISSISSIPPI VALLEY ASSOCIATION OF DENTAL SURGEONS, will hold their ——annual meeting in Louisville on the second Wednesday of September next. Drs. Griffith or Goddard will inform members of the place provided for its daily sessions.

Members will try and be present by ten o'clock, A. M., a full attendance is desired—several reports and addresses are expected and we have no doubt two or three days will be spent very pleasantly and profitably to the members.

The Subscribers to the Register, will please recollect that the present number closes the fourth volume, and that the work cannot be sustained without prompt payment. A few to whom we have sent the fourth volume have sent it back, some without giving us any clue by which to know who they are, or even the office from which it is sent. Subscribers would do well to commence with the beginning of the volume, some wish to commence at first of the years—this is more inconvenient and breaks the sets.

AGENTS FOR THE REGISTER.

*New York.*—Messrs. Jones White & Co.
*Philadelphia.*—Messrs. Jones White & Co.
*New Orleans.*—Messrs. Clark & Ellison.
*St. Louis.*—Drs. Fithian and Spalding.
*Louisville.*—Mr. M' Alister, Druggist.
*Cincinnati.*—Dr. J. M. Brown, Dental Depot.
*Pittsburgh.*—Mr. Dunlevy, Gold Beater.

We have in receipt on exchange the following medical and dental periodicals:—

Western Lancet;
Charleston Medical and Surgical Journal;
St. Louis Medical and Surgical do;
Boston Medical and Surgical do;
The Western Medico and Chirurgical Journal;
American Journal of Dental Science;
Dental Recorder;
Dental News Letter.

We bespeak from the profession short and practical articles on any subject appertaining to the profession, and hope contributors will send in their articles in advance of our publication months, so that the Register may not be delayed in the regular time of publication.

Agents.—We have agents now in Philadelphia, New York, New Orleans, St. Louis, Louisville, Pittsburgh, and Cincinnati, and hope that our subscribers will make it convenient to settle their arrearages as soon as possible—receipts from any of these agents will be good.

The present number has been delayed, expecting two or three articles from contributors, but which have not yet been received.

